# Characterization of pSer129-αSyn Pathology and Neurofilament Light-Chain Release across In Vivo, Ex Vivo, and In Vitro Models of Pre-Formed-Fibril-Induced αSyn Aggregation

**DOI:** 10.3390/cells13030253

**Published:** 2024-01-29

**Authors:** Maja L. Hansen, Malene Ambjørn, Mikkel N. Harndahl, Tau Benned-Jensen, Karina Fog, Kaare Bjerregaard-Andersen, Florence Sotty

**Affiliations:** 1 Neuroscience, Molecular and Cellular Pharmacology, H. Lundbeck A/S, Valby, 2500 Copenhagen, Denmark; hmaj@lundbeck.com (M.L.H.); maam@lundbeck.com (M.A.); kape@lundbeck.com (K.F.); 2 Biotherapeutic Discovery, H. Lundbeck A/S, Valby, 2500 Copenhagen, Denmark; minh@lundbeck.com (M.N.H.); bjer@lundbeck.com (K.B.-A.); 3 Neuroscience, Histology and Pathology Models, H. Lundbeck A/S, Valby, 2500 Copenhagen, Denmark

**Keywords:** alpha-synuclein, pre-formed fibrils, protein aggregation, neurofilament light chain, biomarkers, neurodegeneration, F28tg mouse model, synucleinopathies, Parkinson’s disease

## Abstract

Protein aggregation is a predominant feature of many neurodegenerative diseases, including synucleinopathies, which are characterized by cellular inclusions containing α-Synuclein (αSyn) phosphorylated at serine 129 (pSer129). In the present study, we characterized the development of αSyn pre-formed fibril (PFF)-induced pSer129-αSyn pathology in F28tg mice overexpressing human wild-type αSyn, as well as in ex vivo organotypic cultures and in vitro primary cultures from the same mouse model. Concurrently, we collected cerebrospinal fluid (CSF) from mice and conditioned media from ex vivo and in vitro cultures and quantified the levels of neurofilament light chain (NFL), a biomarker of neurodegeneration. We found that the intra-striatal injection of PFFs induces the progressive spread of pSer129-αSyn pathology and microglial activation in vivo, as well as modest increases in NFL levels in the CSF. Similarly, PFF-induced αSyn pathology occurs progressively in ex vivo organotypic slice cultures and is accompanied by significant increases in NFL release into the media. Using in vitro primary hippocampal cultures, we further confirmed that pSer129-αSyn pathology and NFL release occur in a manner that correlates with the fibril dose and the level of the αSyn protein. Overall, we demonstrate that αSyn pathology is associated with NFL release across preclinical models of seeded αSyn aggregation and that the pharmacological inhibition of αSyn aggregation in vitro also significantly reduces NFL release.

## 1. Introduction

α-Synuclein (αSyn) is an aggregation-prone protein and the prime constituent of fibrillar inclusions, which occupy the cytoplasm of neurons and oligodendrocytes in patients with synucleinopathies such as Parkinson’s disease and multiple system atrophy [1,2,3]. The discovery that αSyn aggregates can spread from inclusion-bearing neurons to grafted cells in humans [4] and mice [5] suggests that αSyn aggregates can transmit themselves from cell to cell, akin to a proteinaceous infectious particle (“prion”). The prion-like spread of misfolded αSyn is believed to accelerate disease progression, as αSyn aggregates internalized by recipient cells can template (“seed”) the misfolding of native αSyn, leading to the amplification of αSyn pathology [6,7,8,9].

The induction of αSyn aggregates can be modeled by adding or injecting αSyn pre-formed fibrils (PFFs) into cell cultures [10] and animals [11], respectively. After their internalization and escape from the endolysosomal system, the PFFs seed the conversion of native αSyn into fibrillar forms [12], resulting in protein- and organelle-rich aggregates that successfully mimic key features of patient-derived Lewy Bodies, such as detergent-insolubility and the phosphorylation of αSyn at serine 129 (pSer129-αSyn) [10,13,14]. In agreement with the seeding-and-spreading hypothesis, the histological analysis of pSer129-αSyn pathology in PFF-injected mice has shown that the spreading pattern of pathological αSyn is dependent on the brain connectome and *Snca* expression, indicating that the spreading of αSyn aggregates is governed by neuronal wiring and the amount of endogenous αSyn available for seeding [15]. Furthermore, the seeded aggregation of αSyn induces cellular deterioration, as evidenced by synaptic dysfunction [10,14,16,17], reduced mitochondrial respiration [14,18], cytosolic Ca^2+^ imbalance [19], impaired organelle trafficking [20,21], and the malfunction of the autophagosomal–lysosomal system [18,22,23].

Mounting evidence suggests that glial cells influence the pathogenesis of synucleinopathies. Microglia, the brain-resident immune cells, exhibit increased activity in Parkinson’s disease patients when compared to healthy controls [24]. Additionally, both in vivo and in vitro data suggest that exposure to misfolded αSyn is immunogenic, leading, among other processes, to inflammasome activation and the secretion of proinflammatory cytokines [25,26,27,28,29]. Histological examination reveals that PFF-induced αSyn aggregation triggers pronounced morphological changes in Iba1^+^ microglia in vivo, which become hypertrophic [28,30] and increase in number [28,31].

αSyn aggregation, neuronal dysfunction, and neuroinflammation are believed to contribute to neurodegeneration. Conventionally, PFF-induced cell death has been estimated in vitro by using neuron-specific methods, e.g., the quantification of the number of surviving NeuN^+^ nuclei [10,17,32] or general cytotoxicity assays, such as lactate dehydrogenase [14,18,32,33] and caspase-3 assays [5,14]. The assessment of neurodegeneration in PFF-treated animals is commonly performed by assessing tyrosine hydroxylase immunoreactivity as a proxy for dopaminergic neuron loss in the substantia nigra pars compacta [11,25,30,34]. However, to evaluate neurodegeneration across the brain, tyrosine hydroxylase stainings are of limited use. Recent publications conclude that quantifications of neurofilament light chain (NFL) in biofluids can be used to sensitively estimate neurodegeneration in rodent models of Creutzfeldt–Jakob disease [35], Huntington’s disease [36], and tauopathies [37], as well as in genetic models of synucleinopathies [38]. Here, we investigate the potential of using NFL as a biomarker of neurodegeneration across in vivo, ex vivo, and in vitro models of PFF-induced αSyn aggregation. Our results indicate that αSyn aggregation is a key factor influencing NFL release in these models and that NFL measurements can be used across different model systems to study the timing and severity of the neurodegenerative response and to evaluate the impact of pharmacological interventions.

## 2. Materials and Methods

### 2.1. Mice

C57BL/6 mice (referred to as wild-type (WT) mice) were purchased from Taconic Denmark. C57BL/6OlaHsd mice with an acquired deletion of the *Snca* gene encoding αSyn [39] (referred to as *Snca* KO mice) were purchased from Envigo Netherlands. The tg(snca)F28Pka mouse model (referred to as F28tg) overexpresses human αSyn under the mouse *Snca* promotor and was created on a C57BL/6J background via pronucleus injection of a DNA sequence encoding human αSyn, as previously described [40]. F28tg mice were maintained and bred at Taconic Denmark.

**Figure 1:**
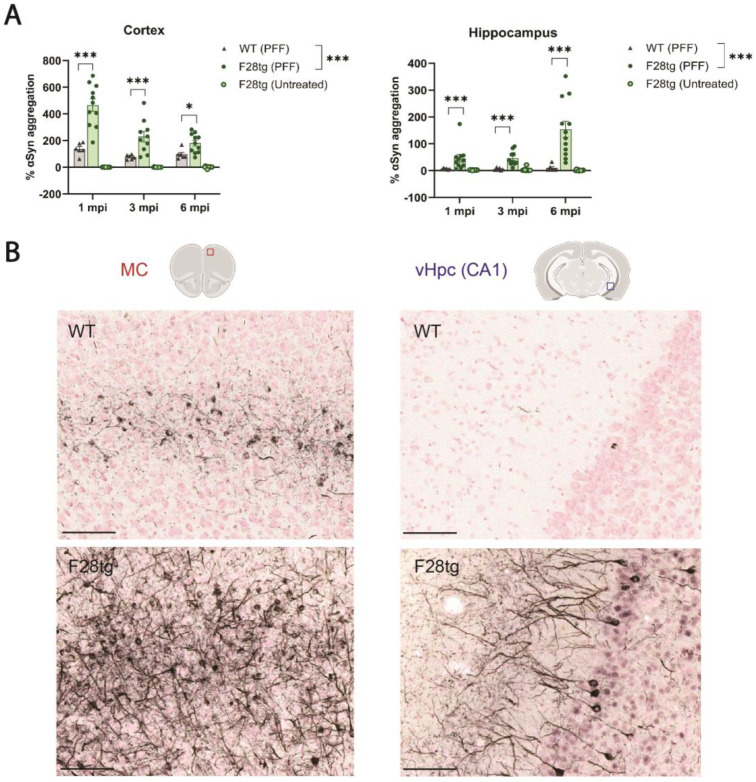
α-Synuclein (αSyn) aggregation in pre-formed fibril (PFF)-injected wild-type (WT) and F28tg mice. (**A**) αSyn aggregation measured by homogeneous time-resolved fluorescence (HTRF) in the cortex and hippocampus of WT (*n* = 6 per time point) and F28tg (PFF: *n* = 10–12 per time point; untreated: *n* = 11–12 per time point) mice at the indicated time points. Bars show mean ± SEM. Statistical analysis was performed on log-transformed data to account for heteroscedasticity using a two-way ANOVA followed by Šídák’s multiple-comparisons test. ns, *p* > 0.05; * *p* ≤ 0.05; *** *p* ≤ 0.001. Only data from PFF-injected animals were included in the analysis. (**B**) Representative histological images of serine-129-phosphorylated (pSer129) αSyn in the motor cortex (MC) and the *cornu ammonis* 1 (CA1) of the ventral hippocampus (vHpc) of WT and F28tg mice at 1.5 mpi. Illustrations showing the approximate location of the imaged brain area were created with BioRender.com. Scale bars represent 100 μm.

**Figure 2:**
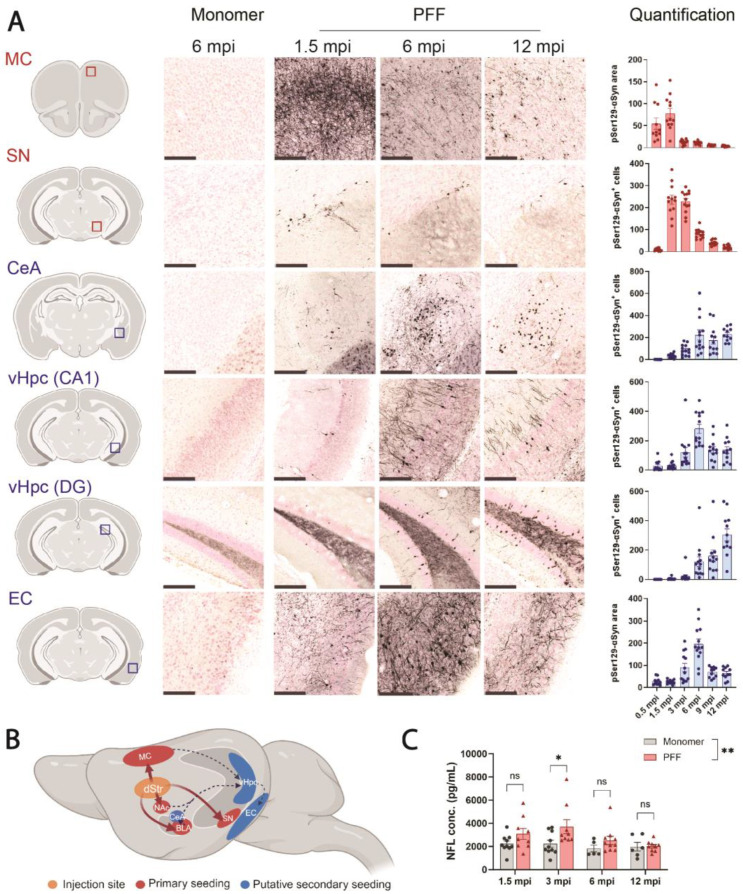
PFF injection induces progressive spread of pSer129-αSyn pathology in F28tg mice. (**A**) pSer129-αSyn pathology in MC, substantia nigra (SN), central nucleus of the amygdala (CeA), CA1 of the vHpc, dentate gyrus (DG) of the vHpc, and entorhinal cortex (EC) at selected time points following striatal injection of αSyn monomers or PFFs. pSer129-αSyn pathology was quantified as area (arbitrary units) of pSer129-αSyn staining (MC, EC) or number of pSer129-αSyn^+^ cells (SN, CeA, CA1, DG) at all time points (months post-injection, mpi), as shown on the right. Graphs show mean ± SEM of *n* = 11–13 mice/condition. Scale bar represents 200 μm. Illustrations on the left indicate the approximate location of the imaged region and were created with BioRender.com, (accessed on 20 October 2023). (**B**) Simplified drawing illustrating how αSyn aggregates might spread from the injection site (dorsal striatum, dStr) to primary seeding regions (red, plain arrows) and secondary seeding regions (blue, dotted arrows). Created with BioRender.com. Abbreviations not already mentioned: NAc, nucleus accumbens; BLA, basolateral amygdala; Hpc, hippocampus. (**C**) Neurofilament light-chain (NFL) levels in cerebrospinal fluid (CSF) from monomer- and PFF-injected F28tg mice at selected time points. Graphs show mean ± SEM of the responses from *n* = 9–10 (PFF) or *n* = 5–10 (monomer) mice per time point. Treatment groups were compared at all time points with two-way ANOVA followed by Šídák’s multiple-comparisons test. ns, *p* > 0.05; * *p* ≤ 0.05; ** *p* ≤ 0.01.

**Figure 3:**
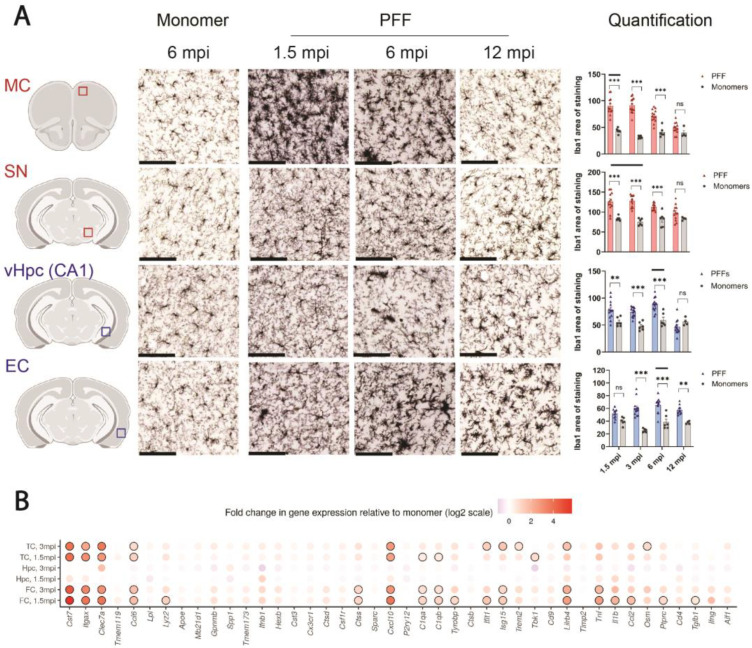
PFF injection induces microgliosis in F28tg mice. (**A**) F28tg mice were injected with αSyn monomers or PFFs into the dorsal striatum, and brain samples were collected for Iba1 staining at intervals ranging from 1.5 to 12 mpi. Displayed are representative Iba1 stainings in the MC, SN, CA1, and EC, along with quantifications of the Iba1-staining area (arbitrary units) in the corresponding regions. Scale bars on images represent 100 μm. Graphs show mean ± SEM of *n* = 11–13 (PFF) or *n* = 4–6 (monomer) mice per group. Black bars on graphs highlight the approximate timing of the observed peak in pSer129-αSyn pathology shown in Figure 2A. For all brain regions, a two-way ANOVA with Šídák’s post hoc test was applied to compare PFF- and monomer-injected animals at each time point. ns, *p* > 0.05; ** *p* ≤ 0.01; *** *p* ≤ 0.001. Illustrations of brain regions were created with BioRender.com. (**B**) Levels of selected inflammation-related genes in the frontal cortex (FC), temporal cortex (TC), and hippocampus (Hpc) measured by Fluidigm in PFF-injected animals when compared to monomer-injected controls (*n* = 6–8 per group). Multiple two-sample *t*-tests with Welch correction were performed on the log-transformed data, and the Benjamini–Hochberg method was applied to control for the false discovery rate (FDR(Q) = 1%). Outlines indicate statistically significant differences (*p* ≤ 0.05).

A total of 40 WT, 205 F28tg, and 10 *Snca* KO mice were used for in vivo experiments. For experiments related to Figure 1A, 12 untreated F28tg, 10–12 PFF-injected F28tg, and 6 PFF-injected WT mice were used per time point. Figure 1B shows representative images from an experiment where 12 WT and 12 F28tg mice were used. The data presented in Figure 2 and Figure 3A are from an experiment including 4–6 monomer-injected F28tg per time point and 11–13 PFF-injected F28tg per time point. Figure 3B shows data from a set of 15 monomer-injected F28tg and 14 PFF-injected F28tg mice (7–8 mice per condition and time point). Finally, the data presented in Figure S2 were obtained from 10 WT and 10 *Snca* KO mice (5 mice per strain and per readout).

All animals had ad libitum access to water and food (Brogaarden, Lynge, Denmark). The light/dark cycle was maintained at 12 h, the room temperature (RT) was 21 ± 2 °C, and the relative humidity was 55 ± 5%. The animal experiments were performed in accordance with the European Communities Council Directive no. 86/609, the directives of the Danish National Committee on Animal Research Ethics, and Danish legislation on experimental animals (license no. 2014-15-0201-00339).

### 2.2. Pre-Formed Fibrils

Human αSyn (uniprot ID: P37840) was expressed in a HEK293 6E cell line and captured on an anion-exchange chromatography column, equilibrated with 20 mM Tris-HCl pH 7.5, directly from the cell media after harvest to avoid any freezing. Elution was performed with a salt gradient to 1 M NaCl over 10 column volumes (CVs). The eluate peak, confirmed by SDS-PAGE to hold αSyn, was subjected to ammonium sulfate (AMS) precipitation by the addition of 40% *w*/*v* AMS for 30 min, followed by 30 min centrifugation at 4500× *g*. The supernatant was discarded. The isolated pellet was solubilized in 20 mM piperazine pH 5.5, and the solution was centrifuged for 30 min at 12,000× *g*. The supernatant was subjected to anion-exchange chromatography and eluted with a NaCl gradient to 500 mM over 10 CVs. The eluate peak was concentrated and loaded onto a Superdex 75 column for size-exclusion chromatography (SEC). The monomeric peak fractions were pooled and aliquoted for storage at −80 °C. Final purity was assessed from SDS-PAGE and analytical SEC. Endotoxin levels were verified to be below 0.5 EU/mg. Furthermore, dynamic light scattering (DLS) was used to verify monodispersity and the absence of aggregated species. The fibrillation was based on the published protocol from Polinski et al. [41]. To prepare PFFs, monomeric αSyn was thawed and concentrated to ~5 mg/mL in dPBS (Gibco, Thermo Fisher Scientific, Roskilde, Denmark). The solution was then transferred to round-bottom tubes, sealed with parafilm, and placed in an orbital shaker at 1000 rpm for 5 days at 37 °C. The assemblies were sonicated on ice using a UP200st Vial Tweeter (Hielscher Ultrasonics, Teltow, Germany) at 100% for a total of 2 min. The temperature of the sample was monitored during sonication, and the process was paused if a temperature of 40 °C was reached. Once the sample was cooled down, sonication continued. A size distribution average (Z-average) below 100 nm was verified by DLS. For representative DLS data from before and after the sonication step, please see Figure S1. The sonicated PFFs were aliquoted in volumes of 10 μL and stored at −80 °C. PFF concentrations are reported in αSyn monomer equivalents.

### 2.3. Stereotaxic Injections

Mice were anesthetized using isoflurane (1.5–2% in 30% oxygen/70% nitrogen) and mounted in a stereotaxic frame while maintaining their temperature at 37 °C via a heating pad. An incision was made in the skin, and a hole was drilled in the skull above the striatum at the following coordinates: AP 0.5 mm anterior to bregma and 2.1 mm lateral to the midline, according to the atlas of Paxinos and Franklin (2001). A calibrated glass capillary (Hirschmann^®^ Ringcaps^®^, 5 μL) was backfilled with PFFs or monomers at a concentration of 2 μg/μL in sterile PBS and lowered in the striatum 2.6 mm below the surface of the brain. A total volume of 2 μL (4 μg) of PFFs or monomers was injected in the right striatum via pressure injection at a rate of 0.3 μL per minute, and the capillary was left in place for an additional 5 min to allow for the diffusion of the material before being removed. The skin was then sutured, and the animals were left to recover from anesthesia before being returned to their home cages. Mice received Temgesic (Bupaq^®^ Vet., buprenorphine 0.03 mg/mL; VetViva Richter GmbH, Wels, Austria) at a dose of 0.1 mg/kg subcutaneously 3 times during the following 24 h for pain relief. Mice were kept undisturbed for the assigned survival time, i.e., 0.5, 1.5, 3, 6, 9, or 12 months post-injection (mpi).

### 2.4. Terminal Sampling Procedures

Mice were anesthetized with Avertin (tribromoethanol, 250 mg/kg i.p.). CSF was collected from the cisterna magna, as described previously [42]. Briefly, mice were placed on a heating pad to maintain their temperature at 37 °C and mounted in a stereotaxic frame with the back of the head facing up and the nose pointing down at ~45°. The dura covering the cisterna magna was exposed and punctured using a pulled, sharpened glass capillary (Hirschmann^®^ Ringcaps^®^, 50 μL) secured in an electrode holder with a 90° bend at the distal end (model 1769, Kopf, Miami, FL, USA). A total of 10–20 μL of blood-free CSF was typically obtained using this method.

For subsequent immunohistochemistry, mice were transcardially perfused with chilled heparinized PBS followed by 4% paraformaldehyde, and their brains were harvested and post-fixed overnight before being transferred to PBS with 0.1% sodium azide.

For the subsequent analysis of αSyn aggregates (homogeneous time-resolved fluorescence) and gene expression (Fluidigm) in brain homogenates, the mice were transcardially perfused with chilled heparinized PBS, and their brains were quickly harvested and dissected to isolate the hippocampus and cortex from the PFF-injected hemisphere. Tissues were snap-frozen on dry ice and stored at −80 °C until homogenization.

### 2.5. Brain Immunohistochemistry

Immunohistochemistry for the visualization of pSer129-αSyn and Iba1 was performed by Neuroscience Associates (Knoxville, TN, USA) using their MultiBrain^®^ technology. The initial optimization of both pSer129-αSyn and Iba1 immunohistochemistry included the testing of the specificity of the stainings by omitting the primary antibody [43]. In addition, a section from a historical study was included in each set of immunostainings to ensure the consistency of the detected signal over time [43]. Briefly, blocks of 25 mouse brains were frozen and sectioned at 35 μm thickness. Free-floating sections were incubated overnight with the primary antibodies (anti-pSer129-αSyn, Abcam, Hong Kong, ab51253; anti-Iba1, Abcam, ab178846). Following rinses, sections were incubated with a biotinylated secondary antibody followed by Vector Lab’s ABC solution (avidin-biotin-HRP complex; VECTASTAIN^®^ Elite ABC, Vector, Burlingame, CA, USA). The sections were again rinsed and then treated with diaminobenzidine tetrahydrochloride (DAB) with nickel and hydrogen peroxide to create a visible reaction product. Following further rinses, the sections were mounted on gelatin-coated glass slides and air-dried. The slides were dehydrated in alcohol, cleared in xylene, and coverslipped.

The quantification of pSer129-αSyn was performed by manually counting the total number of positive cell bodies in all sections spaced 210 μm apart encompassing a given brain area: substantia nigra (SN), central nucleus of the amygdala (CeA), hippocampal *cornu ammonis* 1 (CA1), and dentate gyrus (DG); this resulted in 4 to 15 sections depending on the antero-posterior extent of the area. In the motor cortex (MC) and entorhinal cortex (EC), where neuritic staining was prominent in addition to somatic staining, the total area of positive staining was quantified in 3–4 sections using ImageJ (built-in thresholding analysis).

Iba1-positive staining was quantified as the total area of staining in 3 sections for each brain area using a built-in thresholding analysis in ImageJ bundled with 64-bit Java 8 (Wayne Rasband and contributors, U. S. National Institutes of Health, Bethesda, MD, USA). Before the analysis with ImageJ, all images were converted to 8-bit images.

### 2.6. Organotypic Hippocampal Slice Cultures

Organotypic hippocampal slice cultures (OHSCs) were prepared from post-natal day 6–7 mice. The hippocampi were isolated by dissection, and 350 μm thick hippocampal slices were sectioned with a tissue chopper (McIlwain, Hemmant, Australia) and transferred to a Petri dish containing cold Gey’s balanced salt solution (Sigma, Tokyo, Japan, G9779) with 6.5 mg/L glucose. The separation of the slices was performed with ultra-thin spatulas, and intact slices were transferred onto membrane inserts (Merck, Rahway, NJ, USA, PICM0RG50) floating on top of 1.1 mL/well OHSC plating media (25% heat-inactivated horse serum, 50% OptiMEM with GlutaMAX, 25 mM glucose, 1 mM GlutaMAX, 2.5 mg/L phenol red, 25% HBSS with calcium and magnesium). OHSCs were maintained at 37 °C, 5% CO_2_, and 95% relative humidity. All subsequent medium changes were performed in OHSC maintenance medium (95% Neurobasal medium, 2% B27 supplement, 1 mM GlutaMAX, 25 mM glucose). All medium components were purchased from Gibco, except for glucose and phenol red, which were from Merck and Sigma, respectively. The media were changed on day 3 in vitro (DIV3), on DIV6, and on the day of PFF treatment (DIV9). Seeding was performed by adding 1 μL of PFF solution (5 μg/μL) on top of each slice. After PFF treatment, the media were changed once weekly unless otherwise indicated. During medium changes, the conditioned media were collected and stored at −20 °C until NFL measurements.

The fixation and staining of OHSCs were performed with inspiration from Gogolla et al. [44]. Cultures were fixed for 10 min in 4% paraformaldehyde, washed, and incubated with 20% *v*/*v* methanol in PBS for another 10 min. Membrane inserts with OHSCs were stored at 4 °C in PBS containing 0.02% sodium azide until staining. Cultures were permeabilized overnight (O.N.) in 0.5% TritonX-100 in tris-buffered saline (TBS) and blocked for 6 h at RT in 10% bovine serum albumin (BSA) in PBS. OHSCs were subsequently soaked in a primary antibody (anti-pSer129-αSyn, Abcam, ab51253, 1:1000) solution O.N. The primary antibody solution was removed by three sequential washing steps with 0.3% TritonX-100 in TBS. The secondary antibody (Cy3-donkey-anti-rabbit, Jackson ImmunoResearch Europe Ltd., Ely, United Kingdom, 711-165-152, 1:1000) and DAPI were then applied for 3 h, followed by another 3 washes to remove the excess antibody. OHSCs were cut out of their membrane inserts and mounted on glass slides, coverslipped, and sealed with nail polish. Confocal images were acquired with a Leica DMi 8 microscope equipped with a 5X/0.15 objective (HC PL Fluotar, Leica, Wetzlar, Germany). Images were subsequently imported to Imaris (version 9.0.6, Oxford Instruments, Abingdon, UK), where a mask was designed for the quantification of pSer129-αSyn pathology (Imaris surface analysis with a smoothening value of 1 μm and pixel intensity threshold of 20). OHSCs with abnormal morphology or staining artifacts were excluded from the analysis.

### 2.7. Primary Hippocampal Cultures

Time-mated pregnant female mice were euthanized, and embryos (embryonic day 19) were obtained from their uterine sacs and decapitated immediately. With microscopic guidance, hippocampi were isolated by dissection and stored in cold Hibernate-E (BrainBits). The pooled solution of hippocampi obtained from all embryos in the litter was trypsinized (5 mL 0.05% trypsin-EDTA, 1% penicillin–streptomycin, 99% Hibernate-E without CaCl_2_, 37 °C) for 15 min. Trypsinization was terminated by adding 5 mL of plating medium (10% 10X Minimum Essential Medium, 0.25% GlutaMAX, 0.6% *w*/*v* Glucose, 1 mM sodium pyruvate, 0.22% *w*/*v* sodium bicarbonate, 100 U/mL penicillin–streptomycin, 10% horse serum, all diluted in sterile H_2_O) on top. Hippocampi were then pelleted (300× *g*, 3 min), and the trypsin solution was replaced with 1 mL of neuronal plating medium. The tissue was then resuspended 20 times to mechanically separate the cells. Cells were plated at a density of 4.7∙10^4^/cm^2^ in culture plates (96-well: Greiner, 655090; 6-well: Nunc, 140675) coated with 0.1 mg/mL poly-L-lysine (Merck, P1399). After 3–4 h of incubation, the plating medium was replaced with 100 μL/well neurobasal plus maintenance medium (97.7% Neurobasal PLUS, 0.01 mg/mL Gentamicin, 2% *v*/*v* B27 PLUS supplement, 0.25% *v*/*v* GlutaMAX). After 3 days of incubation, 10 μL/well of cytosine β-D-arabinofuranoside (Sigma) was added to a final concentration of 1 μM to reduce the growth of glial cells. PFFs were diluted in neurobasal plus maintenance medium containing 0.5 μM cytosine β-D-arabinofuranoside and added on DIV5 with a full medium change. All medium components except for glucose (Sigma) and sodium bicarbonate (Sigma) were from Gibco. Experiments with the Hlu-3 antibody targeting human αSyn at amino acids 113–115, as previously described [45], and the isotype control antibody targeting HIV-1 gp120 were performed by pre-mixing the PFFs and the antibodies at the indicated molar concentrations before their addition to the cells on DIV5.

On DIV21, the conditioned medium from the primary hippocampal cultures was collected and stored at −20 °C until NFL measurements, and the cells were washed in PBS and subsequently fixed for 10 min in 4% paraformaldehyde at RT and 10 min in 100% methanol at −20 °C. Blocking was performed in 1% BSA for 1 h, and cultures were subsequently incubated with primary antibodies (anti-pSer129-αSyn, Abcam, ab51253, 1:1000; anti-NeuN, MerckMillipore, Burlington, MA, USA, MAB377, 1:500) for 2 h, washed, and incubated with secondary antibodies (Cy3-donkey-anti-rabbit, Jackson, 711-165-152, 1:1000; Alexa-488-donkey-anti-mouse, Jackson 715-545-150) and Hoechst (Sigma, 63493, 1:500). Unbound antibodies were removed by performing a final washing step (2× in PBS). Cultures were subjected to high-content imaging with a Cellomics ArrayScan^®^ VTI HCS Reader (Thermo Fisher Scientific, Waltham, MA, USA) equipped with a 20×/0.75 objective (Leica). Forty images were acquired per well, and an algorithm (Thermo Scientific HCS Studio: Cellomics Scan Version 6.6.3. Assay template: SpotDetector.v4) that recognizes and records the size and intensity of pSer129-αSyn-positive (pSer129-αSyn^+^) spots was designed and applied to measure the total intensity of pSer129-αSyn^+^ inclusions. Using a different algorithm, we quantified the number of viable (non-pycnotic) NeuN^+^ nuclei per well for the normalization of the pSer129-αSyn signal.

### 2.8. Fractionation by Ultracentrifugation

Cell pellets were collected from 6-well plates on DIV21 and stored at −80 °C until fractionation by ultracentrifugation. Pellets were lysed for 15 min on ice in 1% TritonX-100 in TBS containing protease and phosphatase inhibitors (Roche, Basel, Switzerland), followed by sonication (30 s, 50% amplitude, QSONICA, Newtown, CT, USA Q800R2). To isolate the triton-soluble fraction, lysates were ultracentrifuged (4 °C, 30 min, 100,000× *g*), and the supernatant was collected. The remaining pellet was denatured in 1% SDS diluted in TBS and subjected to sonication and ultracentrifugation, as performed for the triton-soluble fraction. The collected supernatant after the second ultracentrifugation step contained the triton-insoluble and SDS-extractable αSyn. Protein concentrations were measured using the Pierce BCA kit (Thermo Fisher, 23225).

### 2.9. Immunoblotting

The following immunoblotting procedure was applied to protein lysates from primary hippocampal cultures fractionated by ultracentrifugation, as well as samples of recombinant monomeric full-length mouse αSyn (uniprot ID: O55042), monomeric full-length human αSyn (uniprot ID: P37840), and truncated forms (amino acids 1–119 and 1–121) of monomeric human αSyn produced at H. Lundbeck A/S. Samples were prepared for gel loading by mixing with DTT (0.05 M final concentration), loading buffer (Invitrogen, Waltham, MA, USA, NP0007), and MilliQ water. Unless otherwise stated, 2.5 μg/well samples were applied to 4–12% bis-tris gels (Invitrogen, NP0323) soaked in MES running buffer (Invitrogen, NP0002), and electrophoresis was performed at 155 V for ~1 h. Proteins were blotted onto a PVDF membrane (35 V, 90 min) and boiled for 5 min prior to blocking (1 h, LICOR Biosciences, Bad Homburg vor der Höhe, Germany, 927-70001) and incubated with the indicated primary antibodies (anti-mouse αSyn, Cell Signaling, 4179, 1:5000; anti-human αSyn, Invitrogen, MA1-90346, 1:5000; anti-αSyn, Abcam, ab1903, 1:5000; anti-pSer129-αSyn, Abcam, 51253, 1:10000; anti-vinculin, Sigma, V9131, 1:5000; anti-NFL, Invitrogen, 13-0400, 1:5000) O.N. at 4 °C. Membranes were washed 3 times and incubated with the appropriate secondary antibodies (Goat-anti-ms-680, Invitrogen, A21058; Goat-anti-ms-800, LICOR, 926-32210; Goat-anti-rb-800, LICOR 926-32211; Goat-anti-rb-680, Invitrogen, A21077; Goat-anti-rb-680, LICOR 926-68071) 1 h in darkness. After washing, the blots were scanned with an Odyssey Lx imaging system (LICOR).

### 2.10. Neurofilament Light-Chain Immunoassay

MSD plates (MSD, L21XA) were coated with 15 μL/well capture antibody (UmanDiagnostics, Umeå, Sweden, UD1, 1.25 μg/mL in PBS) O.N. at 4 °C. Washing was performed 3 times with 50 μL/well 0.1% Tween-20 in TBS. Wells were blocked for 1 h with shaking (1000 rpm) in 1% casein-PBS (CSF samples) or 3% BSA-TBS (media samples). After washing, CSF samples were diluted 8.3-2.5× (depending on CSF volume) in 1% casein-PBS and applied at 10 μL/well. Medium samples from primary hippocampal cultures and OHSCs were diluted 4× and 2×, respectively, in TBS containing 0.5% BSA and 0.05% Tween-20 and applied at 20 μL/well. The bovine NFL standard (Progen, Heidelberg, Germany, 62008) was diluted in the same buffer as the samples and applied at the same volume. The incubation of samples and the standard was performed for 2 h at RT with shaking. Wells were subsequently washed and incubated with 15 μL/well biotin-labeled detection antibody (UmanDiagnostics, UD3, 0.5 μg/mL). The unbound detection antibody was washed off, and the wells were subsequently incubated for 1 h with 15 μL/well streptavidin-conjugated SULFO-TAG (0.25 μg/mL). Following a wash, the signal was developed by adding 40 μL/well MSD read buffer (MSD, R92TC1) and recorded with an electrochemiluminescence instrument (MSD, SECTOR S600). NFL concentrations in the samples were interpolated from the standard curve (4PL weighted by 1/y^2^) and multiplied by the dilution factor.

### 2.11. Homogeneous Time-Resolved Fluorescence (HTRF)

The indicated brain areas were dissected out and homogenized in lysis buffer (from kit, see below; 18 μL or 9 μL buffer per mg tissue for hippocampal and cortical samples, respectively) supplemented with protease inhibitors (cOmplete, Roche) and phosphatase inhibitors (PhosSTOP, Roche) using Precellys^®^ tubes (CK14) and a Precellys^®^ tissue homogenizer (2 × 50 s, 5000 rpm). The HTRF assay (custom, developed by PerkinElmer Cisbio, Shelton, CT, USA) is based on fluorescence resonance energy transfer between two fluorophores in close proximity coupled to a pair of antibodies recognizing human αSyn at the epitope corresponding to amino acids 126–138, as determined by arrays of overlapping linear peptides at Pepscan (Pepscan Zuidersluisweg 28,243 RC Lelystad, The Netherlands). Using the same monoclonal antibody for the donor and acceptor fluorophores ensures that at least two αSyn molecules must be in close proximity for the assay to give a signal. Samples were diluted 16-fold (cortex) or 8-fold (hippocampus) in lysis buffer prior to mixing with an αSyn antibody mixture containing the antibody coupled to either the Tb-cryptate donor fluorophore (1:20) or the d2 acceptor fluorophore (1:20), as described by the manufacturer. The HTRF signal was measured on a PHEARstar (BMG LABTECH, Ortenberg, Germany) using a 337 nm laser excitation, simultaneous dual emission at 665/620 nm, and HTRF technology. The data are presented as percent αSyn aggregation above background normalized to protein concentrations (μg/μL) measured by the Pierce BCA kit (Thermo Fisher, 23225) according to the manufacturer’s instructions. One data point from the “F28tg cortex 6 mpi” group was excluded from the data analysis, as no protein could be detected by BCA.

### 2.12. Fluidigm

The tissue was homogenized (POLYTRON^®^ PT 1200 E) in 350 μL/sample RA1 buffer (Macherey-Nagel, Dueren, Germany) supplemented with 1% 2-Mercaptoethanol, and the RNA was purified with a NucleoSpin RNA kit (Macherey-Nagel, 740955.250) according to the manufacturer’s instructions. The quality and concentration of RNA in the samples were measured with a Bioanalyzer (Model 2100, Agilent, Santa Clara, CA, USA). Samples with an RNA integrity number ≥ 8 were shipped to Eurofins Genomics (Aarhus, Denmark), where the levels of the indicated genes were measured with a Fluidigm assay. Real-time PCR data were analyzed as described previously [46]. Briefly, Ct values were normalized to the monomer control within each group and the geometric mean of four house-keeping genes: *Actb*, *Eif4a2*, *Atp5b*, and *Sdha*. In addition to the genes mentioned in the figure, we analyzed the expression of *Il10*, *Ifng*, and *Ifnb1*, which were excluded from further analysis, as the expression did not reach the limit of quantification in multiple samples.

### 2.13. Statistics

Statistical analysis methods are stated in the figure legends. *t*-tests and ANOVAs, including the relevant post hoc tests, were performed in GraphPad Prism 9. Linear mixed-effects models and the associated post hoc tests were performed in R using the emmeans (v 1.7.0), lme4 (v1.1.-32), and nlme (v3.1-163) packages.

## 3. Results

### 3.1. Overexpression of Human αSyn Increases αSyn Pathology upon PFF Injection In Vivo

F28tg mice overexpress human *SNCA* under the mouse *Snca* promotor and are therefore expected to overexpress human *SNCA* in brain areas with high *Snca* expression, such as the cortex and hippocampus (Allen Brain Atlas). To assess how the overexpression of human αSyn affects PFF-induced αSyn aggregation, we injected F28tg and WT mice with PFFs and quantified the levels of αSyn species containing ≥2 αSyn molecules (referred to as aggregates below for simplicity) in brain homogenates from the animals using an HTRF assay. In both the cortex and hippocampus, we observed a significantly higher level of αSyn aggregation in the F28tg mice when compared to WT at all time points measured (1, 3, and 6 mpi; Figure 1A). Importantly, the HTRF signal in samples from untreated F28tg mice remained at baseline (Figure 1A), confirming that the observed signal was driven by PFF-induced αSyn aggregation. In line with the HTRF quantification of αSyn aggregates, we observed robust pSer129-αSyn pathology in the MC at 1.5 mpi, which appeared more extensive in F28tg mice compared to WT (Figure 1B). Additionally, in accordance with the HTRF data, pSer129-αSyn pathology was clearly visible in both the soma and neurites of the neurons in the CA1 region of the ventral hippocampus (vHpc) of F28tg mice at 1.5 mpi, while it was sparsely present in WT mice (Figure 1B). We furthermore confirmed that endogenous αSyn is required for PFF-induced αSyn aggregation, as we did not detect any seeding in *Snca* KO mice with either the HTRF assay or pSer129-αSyn stainings (Figure S2). In summary, the data indicates that the overexpression of human αSyn increases seeded αSyn aggregation in both the cortex and hippocampus.

### 3.2. PFF Injection into F28tg Mice Induces the Spread of αSyn Pathology and Release of NFL to the CSF

After demonstrating that PFF-treated F28tg mice exhibit exacerbated αSyn aggregation when compared to WT mice, we next characterized the spreading of αSyn aggregates in the F28tg model by quantifying pSer129-αSyn pathology in brain regions that are either directly or indirectly connected to the site of PFF injection. Neurons that are directly connected to the injection site are defined as located in regions of primary seeding (SN and MC), whereas neurons that are separated from the injection site by at least one synaptic connection are considered located in secondary seeding regions (hippocampal CA1, hippocampal DG, CeA, EC). pSer129-αSyn pathology at secondary seeding sites is hypothesized to result from the prion-like spread of αSyn aggregates from primary seeding sites (Figure 2B) [15].

The qualitative evaluation of pSer129-αSyn pathology in PFF-injected F28tg mice revealed a difference in appearance across brain areas. In regions such as the SN, CeA, and, to some extent, the vHpc, pSer129-αSyn pathology appeared mainly as somatic staining with scarce neuritic staining. In cortical areas, especially in the MC, pSer129-αSyn pathology developed first as dense neuritic staining with rare positive cell bodies, while later time points showed a decline in pSer129-αSyn^+^ neurites, with positive staining restricted mainly to cell bodies (Figure 2A, MC). For this reason, pSer129-αSyn pathology was further quantified as a positive area in cortical regions (MC, EC) and positive cell numbers in other regions. The quantitative evaluation further showed that pSer129-αSyn pathology developed faster in primary seeding regions compared to secondary seeding regions. In the SN and MC, the level of pathology peaked at ~1.5–3 and ~1.5 mpi, respectively, followed by a decline in the number of pSer129-αSyn^+^ cells (SN) and the area of pSer129-αSyn pathology (MC) (Figure 2A). In the secondary seeding regions, we observed a peak in pathology at 6 mpi for some areas (CA1 and EC), while the pSer129-αSyn pathology in the DG and CeA continued to increase for up to 12 mpi (Figure 2A). The order of pSer129-αSyn pathology development is consistent with the sequential spread of αSyn aggregates between interconnected brain regions. Importantly, brains from animals injected with the equivalent amount of monomeric αSyn showed the absence of the pSer129-αSyn signal at all time points after the injection (Figure 2A; the 6 mpi time point is representative for all time points).

We next measured NFL levels in the CSF from PFF- and monomer-injected animals and found that NFL levels were elevated in the CSF of PFF-treated animals at 1.5–6 mpi, reaching statistical significance at 3 mpi (Figure 2C). At 12 mpi, the NFL levels in CSF from PFF- and monomer-injected animals were on par, suggesting that PFF treatment induced the most pronounced neuronal death at earlier time points.

### 3.3. The Progressive Spread of αSyn Is Paralleled by Reactive Microgliosis In Vivo

As microglia are known to rapidly react to extracellular αSyn aggregates and neurodegeneration, we subsequently examined the microglial response in regions of primary and secondary seeding in PFF- and monomer-injected F28tg mice. In regions of primary seeding (SN and MC), microglia appeared hypertrophic with enlarged cell bodies and thick processes, leading to significant increases in Iba1 staining at 1.5–6 mpi compared to monomer-injected mice (Figure 3A). Interestingly, at 12 mpi, the microglial phenotype at primary seeding sites in PFF-treated animals appeared similar to monomer-injected controls (Figure 3A), concomitant with the relatively modest pSer129-αSyn pathological burden observed at this time point (Figure 2A). The quantification of Iba1 staining in the EC (secondary seeding area) showed no significant increase at 1.5 mpi but a significant increase from 3 to 12 mpi (Figure 3A), suggesting that both pSer129-αSyn pathology (Figure 2A) and microgliosis in the EC are delayed when compared to primary seeding regions. We additionally quantified the area of Iba1^+^ cells in the posterior CA1, which is the hippocampal subregion where microgliosis is most evident, and found that the Iba1 area was significantly elevated from 1.5 to 6 mpi, whereafter it dropped to the level in monomer-injected animals (Figure 3A).

To further assess whether PFF-induced αSyn aggregation alters the expression of genes associated with neuroinflammation, we performed high-throughput real-time PCR (Fluidigm) with a selected panel of genes on samples from the frontal cortex (FC), temporal cortex (TC), and hippocampus at 1.5 and 3 mpi. Of note, the FC contains the MC, among other cortical areas, whereas the TC contains areas such as the EC and cortical amygdalar area. In both the FC and TC, we observed robust changes in several genes, including *Cst7*, *Itgax*, *Clec7a*, *Ccl6*, *Cxcl10*, *Tnf*, and *Lilrb4*, some of which were significantly elevated at both 1.5 and 3 mpi (Figure 3B). Interestingly, no significant changes in gene expression were observed in the hippocampus at any of the examined time points (Figure 3B).

### 3.4. PFF-Treated Ex Vivo Organotypic Hippocampal Slices Cultures Develop pSer129-αSyn Pathology and Release NFL in a Time-Dependent Manner

**Figure 4:**
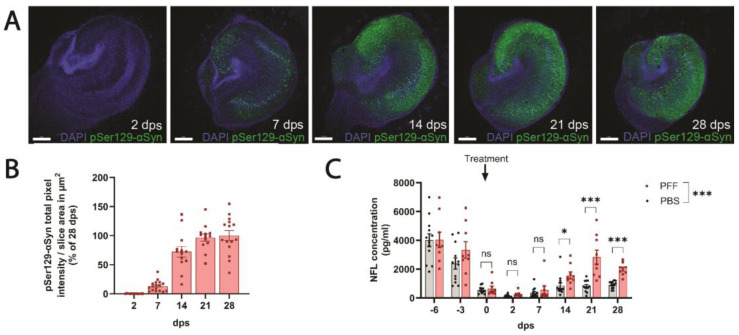
PFF-induced pSer129-αSyn pathology and NFL release occur in a time-dependent manner ex vivo. (**A**) Representative confocal images (scale bars equal 300 μm) and (**B**) image-based quantification of pSer129-αSyn levels normalized to slice area in F28tg organotypic hippocampal slice cultures (OHSCs) at 2–28 days post-seeding (dps) with PFFs. Data from 3 independent experiments with *n* = 3–6 OHSCs per time point is shown. Bars indicate mean ± SEM. (**C**) NFL levels in OHSC-conditioned media. Bars show mean ± SEM of data from 3 independent experiments with *n* = 3–5 wells (each containing 3 OHSCs) per condition. Statistical analysis was performed on the log-transformed 0–28 dps data with a repeated-measures two-way ANOVA followed by Šídák’s multiple-comparisons test. ns, *p* > 0.05; * *p* ≤ 0.05; *** *p* ≤ 0.001.

Ex vivo PFF models offer the advantage of studying the consequences of αSyn aggregation in a semi-realistic three-dimensional environment with a higher throughput and using fewer animals than in vivo studies. Hence, we characterized the progression of PFF-induced pSer129-αSyn pathology and NFL release in OHSCs from F28tg mice. PFF-treated F28tg OHSCs developed pSer129-αSyn inclusions in a time-dependent manner. The first pSer129-αSyn inclusions appeared between 2 and 7 days post-seeding (dps) and plateaued at ~14–21 dps (Figure 4A,B). The quantification of NFL in the conditioned media of the OHSCs showed an initial high level (Figure 4C), likely due to the degeneration associated with the slice preparation, but at the time of the PFF addition, the NFL levels were stabilized at a relatively low level (Figure 4C). At 14–28 dps, the NFL levels were significantly increased compared to those in PBS-treated OHSCs (Figure 4C), indicating a close correlation between the amount of pSer129-αSyn pathology and neurodegeneration.

### 3.5. αSyn Aggregation Is a Key Determinant of NFL Release in Primary Hippocampal Cultures

Inspired by the data from in vivo and ex vivo models, we next investigated whether the NFL assay could also be applied to samples from in vitro primary hippocampal cultures, thereby addressing the current lack of sensitive assays available for quantifying neurodegeneration in vitro.

**Figure 5:**
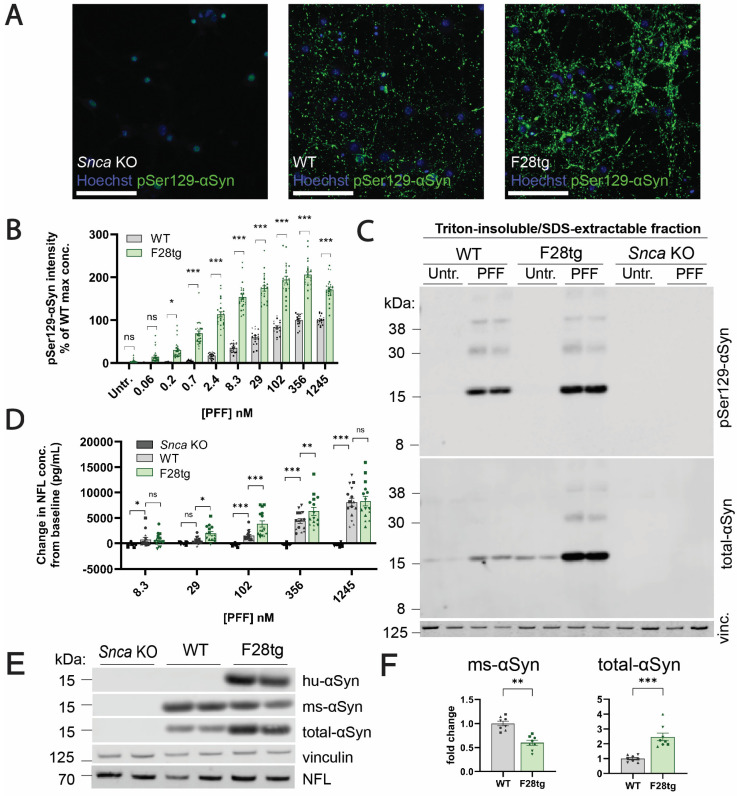
PFF treatment induces different levels of pSer129-αSyn pathology and NFL release in *Snca* KO, WT, and F28tg primary hippocampal cultures. (**A**) Representative Cellomics images (scale bars equal 100 μm) of pSer129-αSyn immunostainings in *Snca* KO, WT, and F28tg cultures exposed to PFFs (29 nM) and (**B**) the corresponding quantifications of pSer129-αSyn pathology per well normalized to the number of NeuN^+^ nuclei and presented as % of the pSer129-αSyn signal in WT cultures that received the highest concentration of PFF. Data are shown as mean ± SEM of 3–5 independent experiments with *n* = 3–6 replicates per condition each. (**C**) Immunoblot showing that pSer129-αSyn and αSyn are enriched in the triton-insoluble/SDS-extractable fractions of F28tg and WT cultures upon PFF treatment (representative of 3–4 independent experiments, *n* = 2 replicates per experiment). (**D**) NFL levels in conditioned media from wells corresponding to the cultures in (**B**). Data are shown as mean ± SEM of 3–5 experiments, each with 3–6 replicates. In each experiment and for each mouse strain, the mean level of NFL in untreated cultures (baseline) was subtracted from the data points. (**E**) Immunoblots showing the levels of human αSyn (hu-αSyn), mouse αSyn (ms-αSyn), total αSyn, vinculin (vinc., loading control), and NFL in the triton-soluble fraction of lysates from untreated (untr.) *Snca* KO, WT, and F28tg cultures (representative of 3–4 independent experiments with *n* = 2 replicates). (**F**) Quantifications of ms-αSyn and total αSyn levels assessed by immunoblotting. Band intensities were normalized to vinculin and plotted as mean fold change ± SEM relative to WT (*n* = 4 experiments, *n* = 2 replicates per experiment). Data points originating from the same experiment have the same shape. Statistics in (**B**,**D**,**F**) were obtained by fitting a linear mixed-effects model including all technical and biological replicates with the experiment number as a random effect, followed by Šídák’s post hoc adjustment (**B**) or Dunnett’s post hoc test (**D**). ns, *p* > 0.05; * *p* ≤ 0.05; ** *p* ≤ 0.01; *** *p* ≤ 0.001.

Consistent with the in vivo observations, F28tg primary hippocampal cultures exhibited the increased formation of αSyn aggregates compared to cultures generated from WT mice following PFF treatment, both when assessed by imaging-based quantification of pSer129-αSyn staining intensity (Figure 5A,B) and when assessed by immunoblotting for the levels of triton-insoluble/SDS-extractable αSyn and pSer129-αSyn (Figure 5C).

In response to seeded αSyn aggregation, F28tg and WT primary hippocampal cultures exhibited a dose-dependent increase in NFL release into the conditioned media (Figure 5D). Of note, the NFL release from F28tg cultures was significantly higher than the release from WT cultures at intermediate PFF concentrations, above which the NFL release from both culture types was similar (Figure 5D). *Snca* KO cultures did not form any pSer129-αSyn^+^ aggregates after seeding (Figure 5A,C) and had significantly lower levels of NFL in the media when compared to WT (Figure 5D), supporting that PFF-induced neurodegeneration is dependent on endogenous αSyn expression. Additionally, we confirmed that the results from the NFL assay were in line with other methods used to quantify cell death or neuronal viability (Figure S3).

To further characterize the αSyn expression in the *Snca* KO, WT, and F28tg primary hippocampal cultures, we performed immunoblotting. Expectedly, the expression of αSyn was absent in *Snca* KO cultures, while the expression of human αSyn was restricted to F28tg cultures (Figure 5E). Using a pan-specific anti-αSyn antibody (clone 4D6, Figure S4D) recognizing a C-terminal epitope (amino acid 124–134 [47], Figure S4E) shared between human and mouse αSyn, we detected a ~2.5-fold increase in total αSyn levels in F28tg in vitro primary hippocampal cultures when compared to WT (Figure 5E,F). Interestingly, the levels of mouse αSyn were ~0.4-fold lower in F28tg cultures when compared to WT (Figure 5E,F), potentially reflecting a compensatory downregulation in response to the αSyn overexpression in F28tg mice.

By blotting for total αSyn, mouse αSyn, human αSyn, and pSer129-αSyn in the triton-insoluble/SDS-extractable fraction of lysates from F28tg primary hippocampal cultures, we additionally confirmed a PFF-dose-dependent increase in aggregate formation contributed by both mouse and human αSyn (Figure S4A,B) and a concomitant reduction in mouse triton-soluble αSyn (Figure S4A,C), highlighting the sequestration of soluble αSyn into insoluble aggregates. We also observed a PFF-dose-dependent increase in triton-insoluble/SDS-extractable pSer129-αSyn, peaking at ~29 nM PFF (Figure S4A,B).

### 3.6. PFF-Induced Neurodegeneration In Vitro Is Inhibited by an Anti-αSyn Antibody

**Figure 6:**
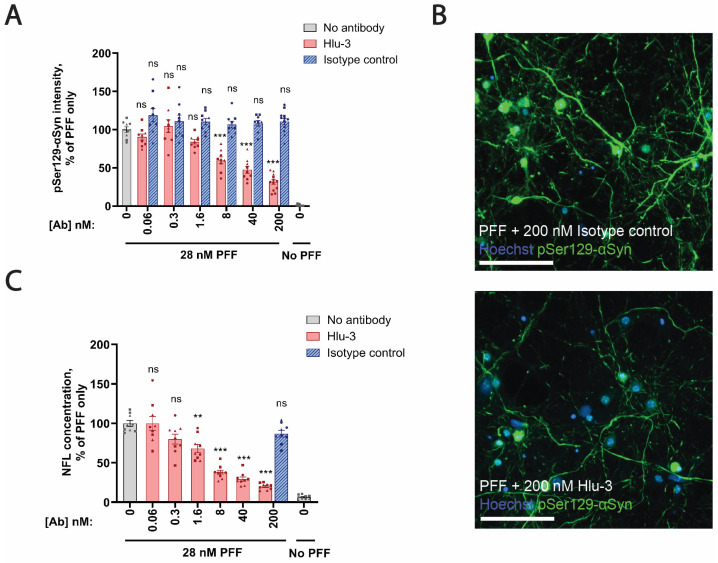
Hlu-3 inhibits seeding and NFL release in PFF-treated F28tg primary hippocampal cultures. (**A**) Quantification of pSer129-αSyn^+^ aggregates by high-content imaging in cultures treated with no antibody, Hlu-3, or an isotype control antibody. pSer129-αSyn levels were normalized to the number of NeuN^+^ nuclei prior to normalization to the “no antibody/PFF only” group in each experiment. The bars show mean ± SEM of data from 3 independent experiments, each with *n* = 3–6 replicates per condition. (**B**) Representative Cellomics images from the cultures treated with isotype control or Hlu-3 antibody in combination with PFF. Scale bar is 100 um. (**C**) Measurement of NFL levels in conditioned media from the cultures in (**A**). The bars show mean ± SEM of data from 3 independent experiments, each with *n* = 3 replicates per condition. Data points originating from the same experiment have the same shape. Statistical analysis in (**A**,**C**) was applied to data from PFF-treated wells using a linear mixed-effects model including all technical and biological replicates accounting for experiment number as a random effect. Before statistical analysis, data in (**C**) were log-transformed to account for heteroscedasticity. Treatment groups were compared to “no antibody/PFF only” with Dunnett’s test. ns, *p* > 0.05; ** *p* ≤ 0.01; *** *p* ≤ 0.001.

We next co-treated F28tg primary hippocampal cultures with the anti-αSyn antibody Hlu-3 in combination with PFFs to evaluate its effect on the seeded aggregation of αSyn and NFL release. Treatment with Hlu-3, but not an isotype control antibody with no affinity toward αSyn, significantly reduced pSer129-αSyn^+^ inclusions (Figure 6A,B). Furthermore, the anti-αSyn antibody, but not the isotype control antibody, significantly reduced NFL release (Figure 6C), indicating that Hlu-3 in this setup protects against pathology development and αSyn-aggregate-driven neurodegeneration.

## 4. Discussion

With the aim of studying αSyn pathology development and neurodegeneration in a model with accelerated αSyn aggregation, we seeded F28tg mice and cultures with PFFs generated from human αSyn. To support the hypothesis that PFF-induced αSyn aggregation is enhanced by the overexpression of human αSyn, we performed HTRF on in vivo samples (Figure 1A) and immunoblotting for the detection of triton-insoluble/SDS-extractable αSyn on in vitro PFF-treated cultures (Figure 5C) using antibodies that are cross-reactive with human and mouse αSyn. With both HTRF and immunoblotting, we observed increased αSyn aggregate formation in F28tg mice and cultures when compared to WT in support of the exacerbated αSyn aggregation being driven by the overexpression of human αSyn. Further supporting this, we detected the increased formation of pSer129-αSyn in the F28tg mice (Figure 1B) and cultures (Figure 5A–C) compared to WT. We speculate that the increased formation of αSyn aggregates in F28tg mice and cultures could be driven by the species compatibility between human PFFs and human αSyn expression, as demonstrated by others [34], and/or the increased levels of αSyn available for PFF-templated aggregation. While our data suggest that the overexpression of human αSyn increases seeded αSyn aggregation, it deserves to be mentioned that the relative quantification of pSer129-αSyn^+^ aggregates in F28tg and WT mice and cultures is limited by potential differences in the affinity of the anti-pSer129-αSyn antibody toward human and mouse pSer129-αSyn, the ability of mouse kinases and phosphatases to regulate the phosphorylation of aggregated human and mouse αSyn, and the susceptibility of human and mouse αSyn to C-terminal truncations that cleave off the pSer129 modification.

Based on the previous literature suggesting that αSyn aggregation causes neuronal deterioration and death [10,14,17], we expected to measure increased NFL release in the seeded F28tg primary hippocampal cultures with a corresponding higher pathology load compared to WT and *Snca* KO cultures (Figure 5A–C). At intermediate PFF concentrations (29, 102, and 356 nM), the NFL levels in the conditioned media were significantly higher in F28tg cultures compared to WT. However, no difference in NFL levels between F28tg and WT cultures was found at the highest PFF concentration (1245 nM) (Figure 5D). As neurons in primary hippocampal cultures continuously develop processes after plating, we speculate, based on our routine inspections of the cells with light microscopy, that the highest PFF concentration interfered with neuronal outgrowth in the F28tg cultures, potentially limiting the amount of NFL released in response to PFF-mediated toxicity. While we did not collect data to support this hypothesis, we suspect that the observed impaired neuritic outgrowth in the F28tg cultures treated with the highest concentration of PFF could be a cellular response to αSyn aggregation, explaining why it was more prominent in the F28tg cultures when compared to WT. Importantly, the NFL level in conditioned media from PFF-treated *Snca* KO cultures, which do not form αSyn aggregates, remained at baseline, further indicating that PFF-induced neuronal toxicity is driven by the aggregation of endogenous αSyn and not mediated by the fibrils per se.

Consistent with the prion-like nature of αSyn aggregates, we observed a progressive spread of pSer129-αSyn pathology in PFF-injected F28tg mice. Expectedly, early pathology was prominent in the primary seeding areas, the MC and SN, peaking at ~1.5 and ~1.5–3 mpi, respectively. Interestingly, pSer129-αSyn pathology declined dramatically after reaching peak levels (Figure 2A). In the MC, where the pSer129-αSyn pathology was quantified by area due to extensive neuritic staining, the decline in the pSer129-αSyn signal could reflect a change in pSer129-αSyn localization, which becomes less neuritic and more somatic with time, leading to an apparent decrease in area. It could, however, also be due to the death of inclusion-bearing neurons, as noted by others [11,34] and supported by the significant ~1.7-fold increase in NFL levels observed at 3 mpi in PFF- versus monomer-injected animals (Figure 2C). Others have reported changes in NFL in the CSF from A30P-αSyn transgenic mice seeded with A30P-αSyn brain homogenate. In the study by Bacioglu et al., NFL in the CSF increased ~400-fold compared to A30P-αSyn mice injected with homogenate from WT mice [38]. While both our study and the data from Bacioglu et al. support the link between αSyn aggregation and neurodegeneration, it remains to be addressed whether the more robust NFL response observed in their A30P-αSyn brain homogenate-seeded A30P-αSyn mice compared to our PFF-seeded F28tg mice is mediated by differences in the mouse strain, the potency of the seeding material, or other technical variations.

In contrast to the relatively modest PFF-induced changes in NFL levels in vivo (Figure 2C), we observed more pronounced effects of PFF treatment on NFL levels in conditioned media from F28tg OHSCs (Figure 4C) and especially F28tg primary hippocampal cultures (Figure 5D). However, a limitation in our ability to compare the NFL results across model systems is that it was not technically possible to apply comparable amounts of PFF per neuron across in vivo, ex vivo, and in vitro systems. Hence, we speculate that the difference in the magnitude of the NFL response across model systems could be mediated by differences in the amount of induced pathology. This notion is further supported by our data from primary hippocampal cultures demonstrating that the level of αSyn aggregation and the neurodegenerative response are dependent on the amount of seeding material added.

Another important difference to consider when studying PFF-induced αSyn aggregation and NFL release using in vivo, ex vivo, and in vitro model systems is the number of glial cells present, as this might significantly impact both the αSyn-seeding process and the neuroinflammatory response and thus neurodegeneration. While in vivo and ex vivo models contain considerable numbers of glial cells, the proliferation of glial cells in vitro is inhibited by the addition of cytosine β-D-arabinofuranoside to enrich for neurons. We hypothesize that the presence of glial cells might have influenced our results in various ways. For instance, it has been shown that glial cells take up and degrade PFFs [48,49], possibly reducing PFF uptake and αSyn pathology in the neurons. On the other hand, microglia secrete proinflammatory factors in response to PFFs, thereby activating astrocytes, which secrete neurotoxic substances [28], potentially promoting NFL release. However, glial cells also clear dying neurons [50], and their phagocytic activity could therefore also limit the release of NFL into the CSF of mice and conditioned media of OHSCs. Whether glial cells promote or halt neurodegeneration and influence the release of NFL remains an open question, although highly relevant given the prominent microgliosis observed in vivo.

Microgliosis in the primary seeding regions of PFF-injected F28tg mice showed an overall good time- and brain-area-dependent correlation with the amount of pSer129-αSyn pathology (Figure 3A), indicating that microglia respond promptly to αSyn aggregation. Interestingly, at secondary seeding sites, we observed significant changes in Iba1 staining prior to extensive pSer129-αSyn pathology development (Figure 3A), potentially reflecting an early inflammatory response toward extracellular misfolded αSyn and/or factors released from the relatively few seeded neurons. To further characterize the inflammatory response in areas of αSyn aggregation, we analyzed the expression of selected neuroinflammation-relevant genes and found that the levels of several transcripts were significantly changed in the FC and TC, but not in the hippocampus (Figure 3B), where reactive microgliosis, evaluated histologically, was overall relatively mild, although significant when restricted to, e.g., the CA1 of the vHpc (Figure 3A). While one limitation of analyzing tissue in bulk is that it does not allow us to assign the changes in gene expression to certain cell types, it is an interesting notion that some of the most significantly changed genes in PFF-injected mice, e.g., *Cst7*, *Itgax*, *Clec7a*, and *Lilrb4*, are found to be expressed by disease-associated microglia in models of Alzheimer’s disease [51,52,53], suggesting that shared microglial responses exist between protein-misfolding diseases. Additionally, the upregulation of *Clec7a*, encoding the pathogen recognition receptor Clec7a, in microglia has recently been shown to promote neuroinflammation in a mouse model of Parkinson’s disease [54], while the roles of *Cst7*, *Itgax*, and *Lilrb4* in relation to Parkinson’s disease remain to be further investigated. Furthermore, we found that *Tnf* was differentially expressed upon PFF treatment, consistent with published in vitro data from primary microglia [28]. Moreover, in agreement with previous in vitro data from astrocytes treated with conditioned media from PFF-treated microglia [28] or factors (Il-1α, C1q, TNFα) [55,56] known to be secreted by PFF-treated microglia [28], we found a strong PFF-induced upregulation of *Cxcl10* in vivo, making it plausible that the observed changes in *Cxcl10* expression, potentially driven by astrocytes, is induced by microglia.

In summary, we systematically evaluated the levels of pSer129-αSyn pathology and NFL release across in vivo, ex vivo, and in vitro models of seeded αSyn aggregation. We show through in vitro and ex vivo experiments that the release of NFL into conditioned media is significantly influenced by the extent of αSyn pathology, which, in turn, is affected by the mouse strain (mouse and human αSyn levels), PFF concentration, and time. Additionally, we detected a slight increase in NFL in the CSF from PFF-treated F28tg mice, reaching statistical significance at 3 mpi, corresponding to the approximate time point of when αSyn pathology dropped after reaching peak levels. Hence, we demonstrated that NFL, a promising biomarker of a variety of neurological disorders [57], can be measured across different preclinical αSyn-seeding models of variable complexity to improve our understanding of αSyn-driven neurodegeneration, as well as pharmacological interventions.

## Data Availability

The dataset is available from the corresponding author upon reasonable request.

## Supplementary Materials

The following supporting information can be downloaded at https://www.mdpi.com/article/10.3390/cells13030253/s1: Figure S1: Exemplary data from PFF characterization by DLS. Figure S2: Seeded αSyn aggregation requires endogenous αSyn expression. Figure S3: PFF-induced cell death measured by different methods. Figure S4: Dose-dependent PFF-induced αSyn aggregation in F28tg primary hippocampal cultures. The following reference is cited in the Supplementary Material: [58].
