# Supplementary material for: Characterization of pSer129-αSyn Pathology and Neurofilament Light-Chain Release across In Vivo, Ex Vivo, and In Vitro Models of Pre-Formed-Fibril-Induced αSyn Aggregation
Source: Cells. 2024 Jan 29;13(3):253. doi: 10.3390/cells13030253 (PMC10854598; doi:10.3390/cells13030253)
Supplement: Supplementary file 1 [file cells-13-00253-s001.zip › cells-2782131-supplementary.pdf]

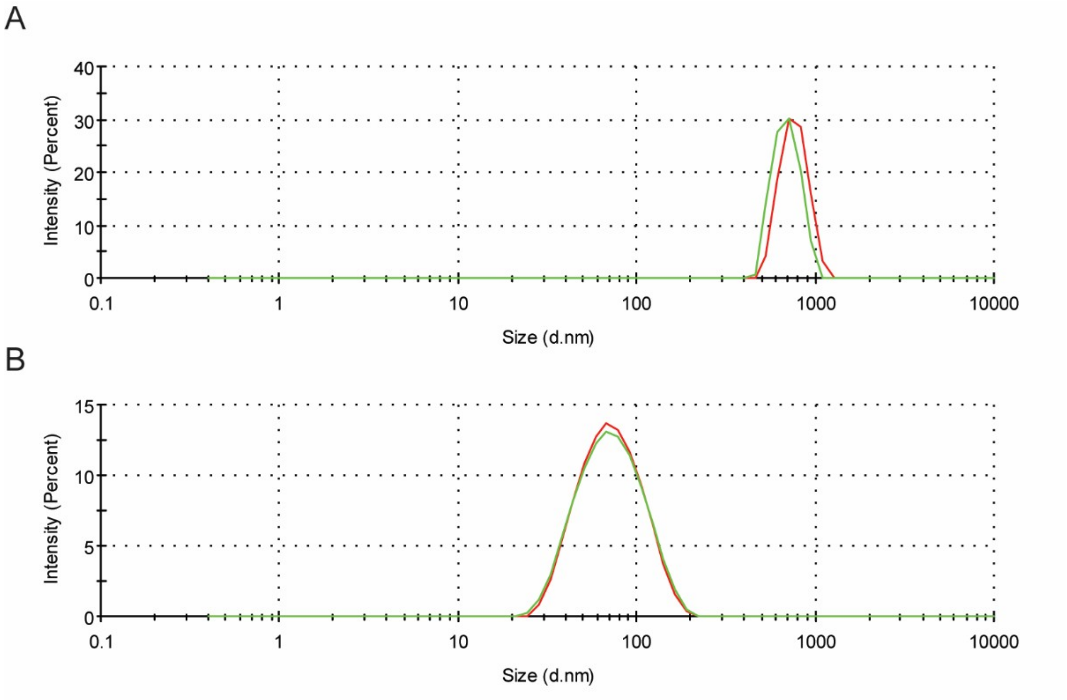

Figure S1. Example on data from PFF characterization by DLS. (a) Non-sonicated PFFs contain large particles corresponding to long or highly clustered PFFs. The distribution is polydisperse and Z-average is 1061 nm. (b) Scheme 65. nm. Red and green curves are technical duplicates.

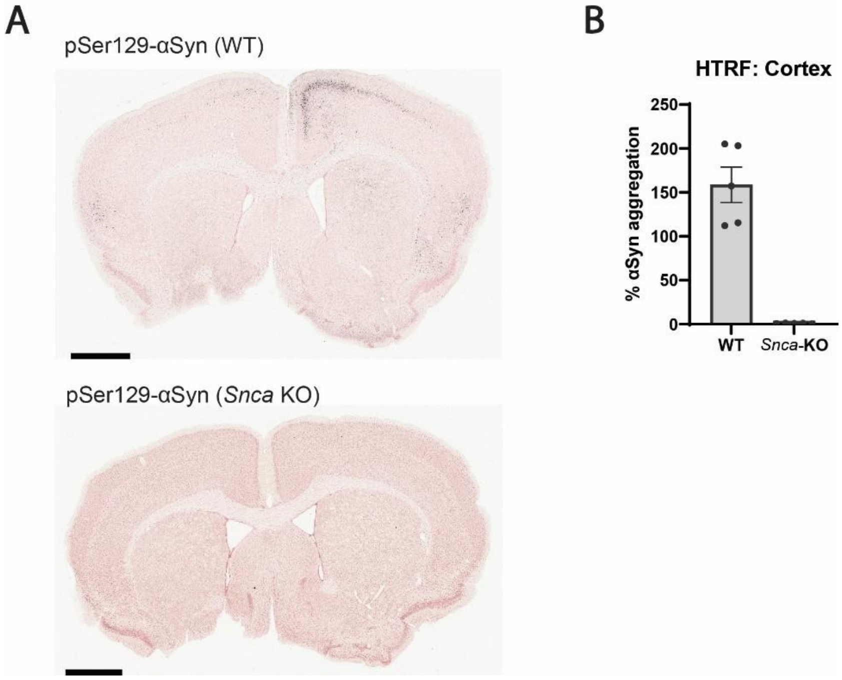

Figure S2. Seeded αSyn aggregation requires endogenous αSyn expression. **(a)** Representative pSer129-αSyn immunohistochemistry in brain sections from WT and *Snca* KO mice (1.5 mpi) injected with PFFs in the right striatum, demonstrating that PFF treatment induces pSer129-αSyn pathology in WT mice, but not in *Snca* KO mice. Scalebars represent 1 mm. **(b)** HTRF data from the cortex of PFF-injected WT and *Snca* KO mice at 1.5 mpi, showing that αSyn aggregation can be detected in PFF-treated WT mice, but not in PFF-treated *Snca* KO mice.

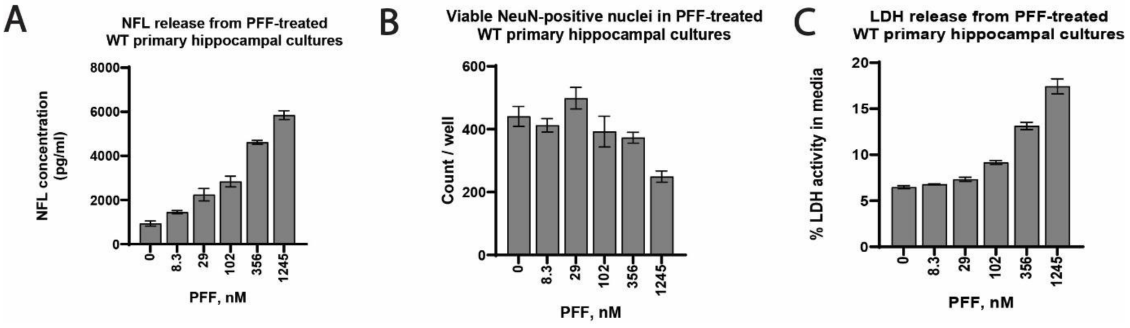

Figure S3. PFF-induced cell death measured by different methods. PFF-induced **(a)** NFL release and **(b)** the corresponding quantification of NeuN-positive nuclei in the cultures. **(c)** Lactate dehydrogenase (LDH) release measurements in WT primary hippocampal cultures from the same experiment (parallel plate) as the cultures in (a-b). LDH measurements were performed with inspiration from Christensen et al (reference [58] in the main manuscript). Briefly, conditioned media and cell lysates were centrifuged at 600 g for 5 minutes, and the LDH activity was measured in the supernatant by mixing the samples (25 µL conditioned media or 7.5 µL cell lysate) with 150 µL/well reaction solution containing 149 µg/mL NADH, 93 µg/mL Na-pyruvate, 0.2 M NaCl, and 0.08 M Tris-base, pH 7.2, 37°C). The absorbance at 340 nm was measured every 20 seconds for 15 minutes with a PHEARstar reader (BMG LABTECH), and the slope of the absorbance change was calculated to measure LDH activity. The LDH activity in the conditioned media is reported as a percentage of the total LDH activity. Data is shown as mean ± SEM and is representative of two independent experiments with n = 3 technical replicates (wells) per condition. Note that the NFL assay appears to be the more sensitive than the LDH assay and the NeuN quantifications.

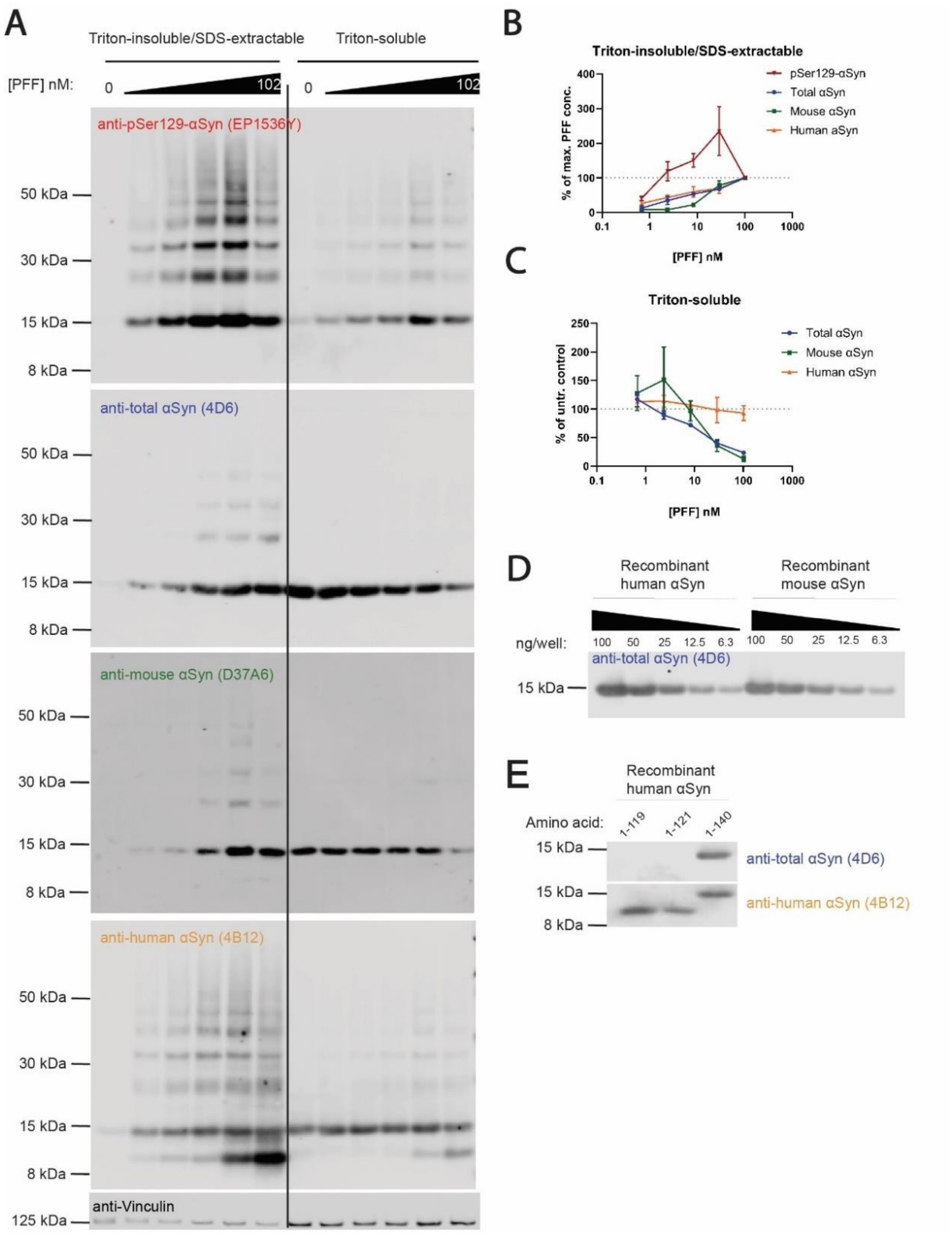

Figure S4. Dose-dependent PFF-induced αSyn aggregation in F28tg primary hippocampal cultures. **(a)** Representative immunoblots and **(b-c)** the corresponding quantifications showing the relative levels of mouse, human, total, and Ser129-phosphorylated αSyn in the triton-soluble and triton-insoluble/SDS-extractable fractions of F28tg primary hippocampal cultures treated with a concentration range of PFFs (3.5-fold dilution starting from 102 nM). The band intensities were normalized to vinculin before normalizing to the reference samples ("102 nM PFF" in figure (b) and "untreated (untr.) control" in figure (c)). Graphs show mean ± SEM of three independent experiments with 1 replicate per condition. (**d)** Immunoblot showing that the anti-total αSyn (4D6) antibody detects both mouse and human recombinant αSyn. **(e)** Immunoblot showing that the epitope of the anti-total-αSyn (4D6) antibody is in the C-terminal end of αSyn, while the epitope of the anti-human αSyn (4B12) antibody detects both full length (amino acid 1-140) and truncated (amino acid 1-119 and 1-121) versions of recombinant human αSyn, explaining why bands corresponding to truncated forms of αSyn (between 8 and 15 kDa markers) are visible in (a) when blotting with the anti-human αSyn (4B12) antibody. In figure (e), 100 ng/well was loaded.
